# Foundational elements of communication in health and medicine: avenues for strengthening the marketing communications mix

**DOI:** 10.1186/s12913-020-05604-9

**Published:** 2020-09-15

**Authors:** James K. Elrod, John L. Fortenberry

**Affiliations:** 1 Willis-Knighton Health System, 2600 Greenwood Road, Shreveport, LA 71103 USA; 2 LSU Shreveport, 1 University Place, Shreveport, LA 71115 USA

**Keywords:** Marketing, Marketing communications, Promotion, Hospitals, Healthcare

## Abstract

**Background:**

When one thinks of opportunities to engage patients, the marketing communications mix often is the first thing that comes to mind. Its five components of advertising, personal selling, sales promotion, public relations, and direct marketing represent tried and true pathways for establishing productive dialogues with customers of healthcare institutions. But in formulating and deploying the marketing communications mix, health and medical establishments must not neglect foundational elements which play vital communicative roles, impacting the perspectives of patients and influencing associated patronage.

**Discussion:**

Many things communicate on behalf of healthcare organizations, including the people employed by them, the places in which they deliver services, and the brands that represent them. As foundational elements of communication, these must be addressed prior to formulating the marketing communications mix, as they influence and impact an institution’s entire communicative potential. Their initial development, however, is just the beginning, as these elements must be managed and maintained continually over the course of organizational life. This article profiles foundational elements of communication and discusses their importance in healthcare marketing, generally, and marketing communications, specifically, providing useful insights for maximizing communicative synergies.

**Conclusions:**

Given the importance of engaging current and prospective patients, healthcare establishments must take steps to ensure exceptional prowess in this area, with communicative skills and abilities being of paramount importance. Proficient deployment of the marketing communications mix is essential, but healthcare providers must also be certain to direct attention toward foundational elements, ensuring that given institutions realize their full communicative potential.

## Background

Health and medical establishments have intensive needs to communicate with current and prospective patients and, in their efforts to connect with their desired audiences, they often turn to the marketing communications mix [1–5]. Classically illustrated, the marketing communications mix contains five principal avenues of communication; namely, advertising (i.e., the paid use of mass media to deliver messages), personal selling (i.e., the use of sales agents to personally deliver messages), sales promotion (i.e., the use of incentives, such as contests and free giveaways, to encourage patronage), public relations (i.e., the use of publicity and other unpaid promotional methods to deliver messages), and direct marketing (i.e., the delivery of messages via mail, the Internet, and similar routes directly to consumers) [2, 6]. Healthcare organizations evaluate each option and select one or more believed to be most capable of reaching target audiences, all for the purpose of enticing patronage or compelling some other form of meaningful exchange [2, 7].

**Fig. 1:**
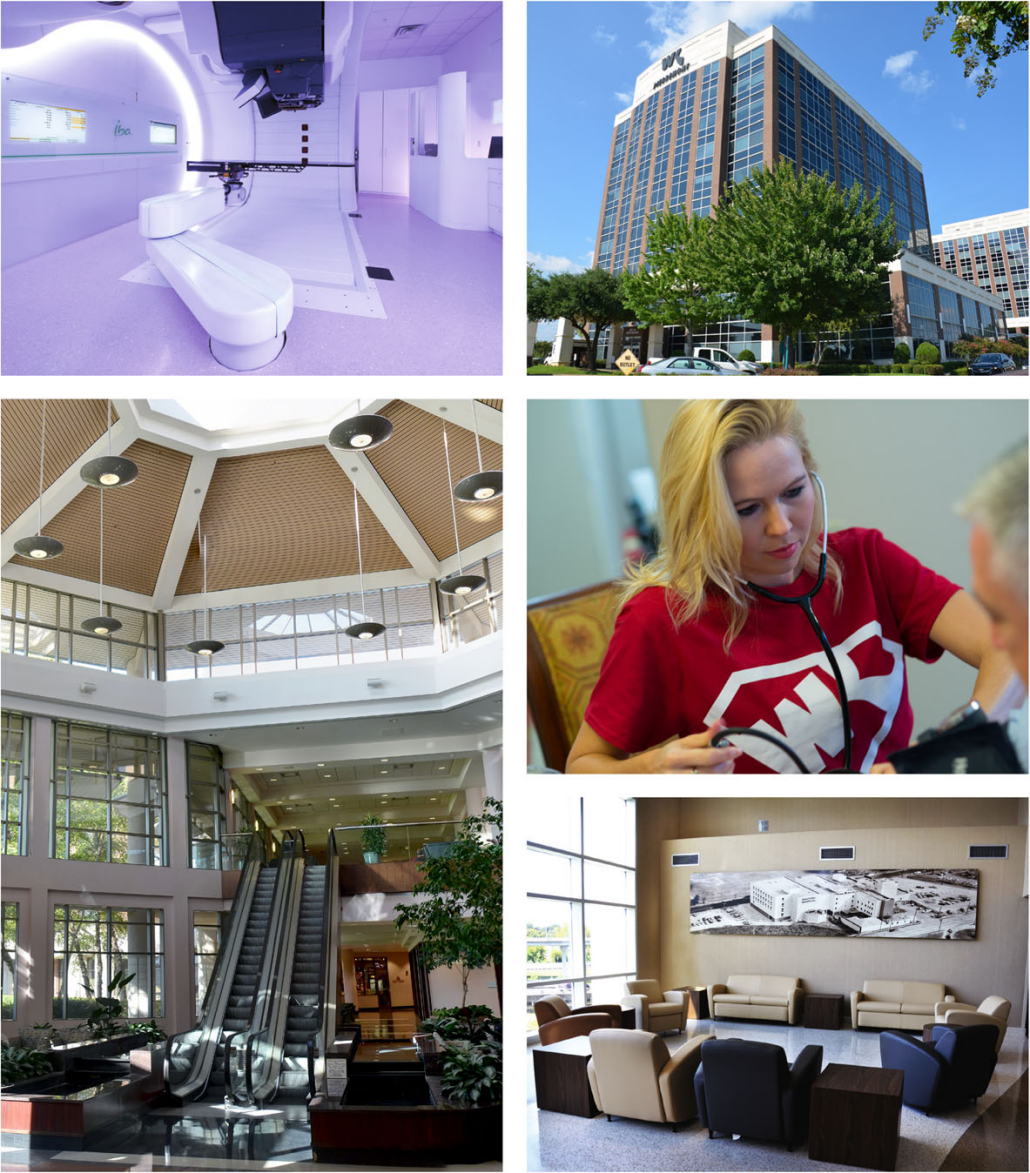
Sights and scenes from Willis-Knighton Health System demonstrating the communicative potential of people, places, and things

**Fig. 2:**
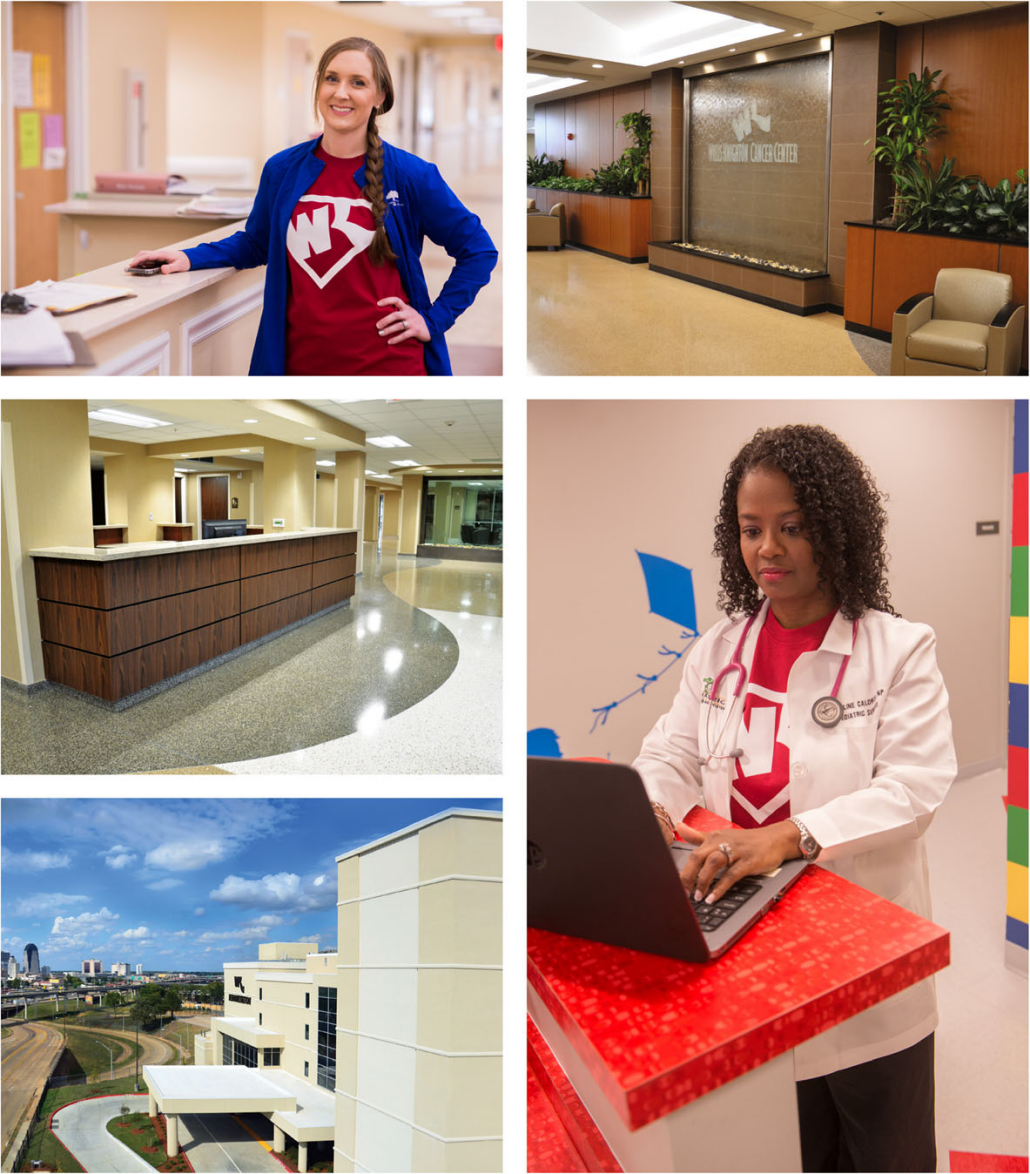
More sights and scenes from Willis-Knighton Health System demonstrating the communicative potential of people, places, and things

But in seeking to engage patients via the components of the marketing communications mix, healthcare establishments must take care to ensure that they do not neglect the bigger communicative picture. In some shape, form, or fashion, virtually everything communicates [2, 6, 8–12]. A healthcare facility’s brand, the care of its buildings and grounds, the state of its service environment, the presentation and conduct of its staff members, and so on convey volumes of information to others, crucially impacting the perspectives of patients and heavily influencing their patronage decisions [2, 6, 8, 13–16]. Such communicative potential is evident from even casual observation, as Figs. 1 and 2, profiling sights and scenes in and around Willis-Knighton Health System, demonstrate. Positive attention directed toward foundational elements affords communicative bedrock; a solid platform for launching components of the marketing communications mix. If addressed improperly, however, no amount of advertising, personal selling, public relations, or any other form of promotion can make up for associated deficiencies, ultimately diminishing opportunities to engage and acquire patients.

While addressing institutional communicators might seem obvious, the rush to telegraph information to outside parties via the marketing communications mix sometimes can cloud priorities, ultimately harming the overall communicative potential of given health and medical establishments [2, 6]. To help ensure that healthcare providers do not lose focus in their quests to proficiently engage patients, this article profiles foundational elements of communication and discusses their importance in healthcare marketing, generally, and marketing communications, specifically, providing useful insights for maximizing communicative synergies.

## Definition and overview

Foundational elements of communication can be described as things that project information to outside parties, but fall outside of the components of the traditional marketing communications mix. Needless to say, this covers significant territory, with these elements including virtually anything and everything that a patient can be exposed to when circulating in or around given healthcare establishments (e.g., adequacy of parking, helpfulness of signage, courtesy of personnel, depth and breadth of services, availability of value-added amenities, comfort of accommodations, quality of food). Viewed through the lens of marketing, these foundational elements represent cornerstones of communication, as they influence conveyances expressed through advertising, personal selling, sales promotion, public relations, and direct marketing [2, 13, 15]. These institutional communicators must be addressed proficiently in advance of formal marketing communications initiatives, ensuring that when prospects are drawn to given healthcare establishments, their expectations built up by deployments of the marketing communications mix are realized. Far from being a static activity, such promotions-critical elements must be managed and maintained continually over the course of organizational life in order to achieve the best marketing communications outcomes [2, 8, 17–21].

Over its many decades of service, Willis-Knighton Health System has never lost sight of the critical need to successfully communicate with audiences. Based in Shreveport, Louisiana and situated in the heart of an area known as the Ark-La-Tex where the states of Arkansas, Louisiana, and Texas converge, Willis-Knighton Health System holds market leadership in its served region where it delivers comprehensive health and wellness services through multiple hospitals, numerous general and specialty medical clinics, an all-inclusive retirement community, and more. The system’s achievement of market leadership is attributed, in part, to communications prowess, permitting Willis-Knighton Health System to effectively engage current and prospective patients, evoking interest and attention, ultimately leading to burgeoning patient volume and customer loyalty [1, 8, 14]. In its pursuit of excellence in formulating and implementing the marketing communications mix, Willis-Knighton Health System has remained mindful of all-important foundational elements of communication which influence and impact aggregate communicative potential and performance [8, 13, 14]. These institutional communicators—categorized per Willis-Knighton Health System’s typology as people, places, and things—are described below.

## People

In many respects, the employees and other personnel serving on behalf of health and medical facilities collectively constitute the most notable communicator for their given institutions. As service entities, interactions between staff members and customers are frequent and intensive, elevating needs for healthcare establishments to ensure that their representatives perform competently and present themselves professionally. Appearance is especially important, meaning that everything from grooming to attire should be in order, with formal dress codes being essential to ensure the conveyance of desirable images to audiences.

Actions also are vital. Beyond conducting assigned duties and responsibilities proficiently, customer service skills are critical, with formal training programs and related initiatives being most helpful in compelling proper mannerisms and behaviors which patients and other parties will find appealing, further reinforcing desired images. Additional conveyances apprising employees of major strategic initiatives, too, are helpful in fostering the communicative potential of staff members. For example, Willis-Knighton Health System actively informs employees of its marketing communications campaigns prior to launch, introducing them to these new initiatives via educational seminars. The institution even holds celebratory launch events, handing out T-shirts, bumper stickers, and related thematic gifts to build employee excitement, morale, and pride. This investment yields educated, informed, and motivated employees, fostering ambassadorship and its associated communicative benefits.

Although people-related matters are well outside of marketing communications, personnel clearly impact a healthcare institution’s ability to attract and engage audiences, thus warranting intensive efforts to ensure that they convey positive images. If this can be arranged, then audiences presenting at healthcare establishments, courtesy of advertising and other outward marketing communications, will be greeted by staff members capable of reinforcing their initial patronage decisions, setting the stage for enduring patient relationships and loyalty [8, 15, 16].

## Places

First impressions are powerful and enduring, with the state of health and medical service environments arguably presenting the initial opportunity for customers to form such impressions. On visiting the campuses of given healthcare facilities or even merely driving by them, many things speak to audiences. The attractiveness of buildings, their state of repair, the care of grounds, the adequacy of parking, the helpfulness of signage, and related exterior aspects communicate boldly to current and prospective patients. A quality exterior certainly can foster notions that given establishments are well operated, supporting perspectives that healthcare services might be delivered with just as much care and attention. The same can be said of interior elements, such as the appearance and comfort of waiting areas, the cleanliness of patient rooms and other spaces, the presentation and functionality of furnishings and fixtures, and related items, with observations of attention to detail here naturally being extrapolated by audiences to other areas of operation.

Since place-related matters indeed have the potential to impact the patronage decisions of patients, healthcare providers must direct extensive attention toward designing and maintaining exterior and interior features to ensure that they match the quality of care provided by given healthcare organizations. Willis-Knighton Health System, for example, invests heavily in the design and upkeep of its buildings and servicescapes to ensure that they are appealing, properly representing the care delivered by the institution. Failures here will erode patient acquisition potential and thwart even carefully devised and successful marketing communications campaigns. However, proper investments and attention directed toward place-related attributes will work synergistically with excellent marketing communications deployments to attract and capture all-important patient volume [8, 15, 16].

## Things

Items not falling in people and places categories are ideal for situating in the things category, permitting the typology to accommodate most any foundational element of communication. The method and manner for populating this category is up to given healthcare institutions. Some establishments might, for example, place technology here, whereas others might consider technology to be a component of servicescapes, making it better suited as a place matter. While such decisions rest with given healthcare entities, one particular item is especially well suited for this category: brands.

Brands are names, logos, slogans, and related elements of identity used by institutions and their product offerings for the purpose of conveying desired images to target audiences. Brands present imagery designed to resonate with customers, enticing patronage, and they also permit audiences to distinguish institutions and their products from competitive offerings, facilitating product differentiation. As brands are featured in virtually every marketing communication forwarded by healthcare entities, care must be taken to ensure that associated identities are meaningful and relevant, as this will have a direct impact on advertising, public relations, and other components of the marketing communications mix. This knowledge led Willis-Knighton Health System to direct extensive resources toward branding, ultimately affording one of the most widely recognized logos in the marketplace. Once thoughtfully prepared, brand-related efforts do not cease; they continue indefinitely as a means of ensuring that formulated identities remain on point. Willis-Knighton Health System, for example, reviews its brand identity on a regular basis, making alterations as needed to ensure desired applicability and impact [8, 13]. While the things category can encompass myriad elements, as deemed suitable by given healthcare establishments, brands certainly should not be overlooked as a key institutional communicator upon which forthcoming marketing communications plans are built.

## Operational reflections

As for operationalizing this typology, given that these elements are foundational in nature and impact the potential of advertising, personal selling, direct marketing, and other promotional deployments, Willis-Knighton Health System recommends conducting readiness assessments in advance of launching marketing communications campaigns. Such readiness assessments involve comprehensively examining the healthcare service or services to be featured in forthcoming campaigns to ensure that audiences responding to given appeals are met with people, places, and things that foster satisfaction and facilitate patronage. These readiness assessments usually involve comprehensive walk-throughs in and around healthcare facilities, typically conducted by institutional leaders, to ensure that service environments are appealing and inviting, examining, for example, the cleanliness of the premises, the adequacy of parking and directional signage, the ease of scheduling appointments, the appearance and professionalism of employees, and so on.

Campaign readiness assessments sometimes are enhanced by the use of mystery shoppers hired to pose as patients, visit designated service environments, make associated inquiries, and report their findings, affording helpful external perspectives which might reveal improvement opportunities otherwise overlooked by personnel. Oversights can and do occur, given institutional and environmental complexities, warranting preventive measures to ensure top performance. The campaign readiness assessment is one such preventive measure that offers health and medical providers one final opportunity before promotions ensue to perfect service experiences, something which can bolster the abilities of institutions to capture patronage and generate positive word-of-mouth communications from satisfied customers. What’s more, implementation is relatively burden-free, with perhaps its simplest application involving completion of a checklist prior to the authorization of marketing communications campaigns.

## Conclusions

Given the importance of engaging current and prospective patients, healthcare establishments must take steps to ensure exceptional prowess in this area, with communicative skills and abilities being of paramount importance. Proficient deployment of the marketing communications mix is essential, but healthcare providers must also be certain to direct attention toward foundational elements that speak boldly on behalf of healthcare institutions, influencing and impacting outwardly-directed marketing communications. Categorized conveniently, comprehensively, and memorably as people, places, and things, these foundational elements must be perfected in order to allow the components of the marketing communications mix to be deployed in a manner to realize their full communicative potential.

## Data Availability

Not applicable.
